# Effects of Prolonged Exposure to Hypobaric Hypoxia on Oxidative Stress, Inflammation and Gluco-Insular Regulation: The Not-So-Sweet Price for Good Regulation

**DOI:** 10.1371/journal.pone.0094915

**Published:** 2014-04-14

**Authors:** Mario Siervo, Heather L. Riley, Bernadette O. Fernandez, Carl A. Leckstrom, Daniel S. Martin, Kay Mitchell, Denny Z. H. Levett, Hugh E. Montgomery, Monty G. Mythen, Michael P. W. Grocott, Martin Feelisch, for the Caudwell Xtreme Everest Research Group

**Affiliations:** 1 Human Nutrition Research Centre, Institute for Ageing and Health, Newcastle University, Newcastle on Tyne, United Kingdom; 2 Warwick Systems Biology Centre, University of Warwick, Coventry, United Kingdom; 3 Warwick Medical School, University of Warwick, Coventry, United Kingdom; 4 University of Southampton, Clinical & Experimental Sciences, Faculty of Medicine, Southampton General Hospital, Southampton, United Kingdom; 5 Centre for Altitude Space and Extreme Environment Medicine, Portex Unit, UCL Institute of Child Health, London, United Kingdom; 6 Division of Surgery and Interventional Science, University College London, Royal Free Hospital, London, United Kingdom; 7 University Hospital Southampton NHS Foundation Trust, Southampton General Hospital, Southampton, United Kingdom; 8 Southampton NIHR Respiratory Biomedical Research Unit, Southampton, United Kingdom; Pennington Biomed Research Center, United States of America

## Abstract

**Objectives:**

The mechanisms by which low oxygen availability are associated with the development of insulin resistance remain obscure. We thus investigated the relationship between such gluco-insular derangements in response to sustained (hypobaric) hypoxemia, and changes in biomarkers of oxidative stress, inflammation and counter-regulatory hormone responses.

**Methods:**

After baseline testing in London (75 m), 24 subjects ascended from Kathmandu (1,300 m) to Everest Base Camp (EBC;5,300 m) over 13 days. Of these, 14 ascended higher, with 8 reaching the summit (8,848 m). Assessments were conducted at baseline, during ascent to EBC, and 1, 6 and 8 week(s) thereafter. Changes in body weight and indices of gluco-insular control were measured (glucose, insulin, C-Peptide, homeostasis model assessment of insulin resistance [HOMA-IR]) along with biomarkers of oxidative stress (4-hydroxy-2-nonenal-HNE), inflammation (Interleukin-6 [IL-6]) and counter-regulatory hormones (glucagon, adrenalin, noradrenalin). In addition, peripheral oxygen saturation (SpO_2_) and venous blood lactate concentrations were determined.

**Results:**

SpO_2_ fell significantly from 98.0% at sea level to 82.0% on arrival at 5,300 m. Whilst glucose levels remained stable, insulin and C-Peptide concentrations increased by >200% during the last 2 weeks. Increases in fasting insulin, HOMA-IR and glucagon correlated with increases in markers of oxidative stress (4-HNE) and inflammation (IL-6). Lactate levels progressively increased during ascent and remained significantly elevated until week 8. Subjects lost on average 7.3 kg in body weight.

**Conclusions:**

Sustained hypoxemia is associated with insulin resistance, whose magnitude correlates with the degree of oxidative stress and inflammation. The role of 4-HNE and IL-6 as key players in modifying the association between sustained hypoxia and insulin resistance merits further investigation.

## Introduction

Ascent to high altitude is associated with a fall in barometric pressure, and with it a reduction in oxygen availability (‘hypobaric hypoxia’). Such exposure, whether in the short- or long-term) leads to diverse endocrine responses (e.g. in insulin signalling, thyroid function and sympatho-adrenal activity [1]–[12]).

Hypobaric hypoxia may also affect the gluco-insular axis. However, data have been inconsistent or contradictory [3]–[6], [12]–[14]: beneficial effects of hypoxia on peripheral insulin action and body weight regulation have, for instance, been suggested [5], [6], [15] whereas a deterioration of insulin signalling has been reported in other studies [3], [7]. The source of such conflicting data may relate to inter-study differences in 1) sample size, 2) intensity of exposure to hypoxia (rate of ascent and maximum height gained: ascent profile) 3) duration of exposure to hypoxia, 4) subjects' phenotypic characteristics, 5) exposure to other environmental stressors (e.g. physical exertion and altered energy balance), and 6) testing conditions (chamber, high-altitude) [16]. Operation Everest II (simulated ascent of Mount Everest over 40 days in a hypobaric chamber) tried to obviate the effect of some of these confounders, with homogenous ascent profile, diet and exercise regime. A key finding was that some endocrine responses were more marked during the last week of the intervention when subject were exposed to the lowest oxygen concentrations [14], [17]. Of note, insulin concentrations at the end of the study were typically 2-fold greater than those at the start, while glucose levels remained unaltered, suggesting the development of insulin resistance [14].

Such findings are of possible importance to the pathogenesis of disease at sea level: there is renewed interest in the role of chronic hypoxia as a potential causative factor in the pathogenesis of insulin resistance. Indeed, chronic intermittent hypoxia (CIH) due to obstructive sleep apnea (OSA) may contribute to the development and progression of insulin resistance and diabetes [18]–[21]. OSA appears to be a predictor of abnormal glucose metabolism in chronically sleep deprived obese adults [19].

Such effects on insulin resistance may be specifically mediated through the genesis of associated adipose tissue hypoxia (ATH) [22], This, in turn, may related to the occurrence of enhanced adipocyte growth, which may not be accompanied by a parallel, functional expansion of stromal and vascular tissue to adequately satisfy the nutritive requirements of the newly formed tissue [23]–[25]. A consequent decrease in convective and diffusive oxygen flux may result in reduced cellular oxygen availability leading to a progressive impairment of oxidative energetic reactions [26]. Prolonged cell/tissue hypoxia induces adaptive mechanisms including production of reactive oxygen species (ROS), inflammatory adipokines and adipose tissue macrophage infiltration. In addition, hypoxic exposure may also affect gluco-insular homeostasis by increasing systemic inflammation [27] and modification of pancreatic β-cell function [28].

Progressive exposure of human volunteers to hypobaric hypoxia at high altitude provides an opportunity to explore associations between hypoxia, oxidative stress, inflammation and gluco-insular deregulation as well as to identify novel mechanistic targets. The 2007 Caudwell Xtreme Everest (CXE) expedition [16] provided such an opportunity. We evaluated whether sustained hypoxemia was associated with alterations in glucose and insulin homeostasis. Specifically, patterns of change in indices of insulin resistance (homeostasis model assessment of insulin resistance, HOMA-IR) [29], insulin secretion (C-Peptide) and hepatic insulin extraction (C-Peptide/Insulin Ratio) [30] were evaluated. In addition, we investigated the role of oxidative stress (Isoprostanes (8-iso-prostaglandin F-2α) and 4-hydroxy-2-nonenal (4-HNE), Total Glutathione, Reduced Glutathione (GSH), Oxidized Glutathione (GSSG)) and inflammatory biomarkers (Interleukin 6 (IL-6), C Reactive Protein (CRP), Macrophage Migration Inhibitory Factor (MIF), Tumour Necrosis Factor Alpha (TNF-Alpha) and counter-regulatory hormones (Glucagon, Adrenalin, Noradrenalin) as putative causal factors linking hypobaric hypoxia to changes in gluco-insular action.

## Methods

The study was approved by the University College of London (UCL) Research Ethics committee, in accordance with the Declaration of Helsinki. Verbal and written informed consent was obtained from all subjects. The study took place between January and June 2007.

### Subjects

Twenty-four healthy subjects (18 male; mean age 35.2 y; range 19–59 y) who were investigators on the CXE 2007 research expedition to Mount Everest participated in this study [16]. All subjects were sea level natives free of cardiovascular or respiratory disease and took no medication. Criteria for participation have been described elsewhere [16].

### Study Protocol

The design and conduct of this study is described elsewhere [16]. Briefly, subjects underwent baseline testing in London (altitude 75 m) before travelling by plane to Kathmandu, Nepal (1,300 m). From there they flew to Lukla (2,800 m) on expedition day 1 and then trekked to Everest Base Camp (EBC; 5,300 m), arriving on expedition day 13. The ascent profile is detailed in **Table S1 in File S1**. Testing was repeated during the ascent in field laboratories in Kathmandu (1,300 m; day −3 to 0), Namche (3,500 m; day 4 to 6), Pheriche (4,250 m; day 9 to 10), and at EBC (5,300 m; day 15 to 17, EBC week 1). The subjects were then divided into two subgroups: Group 1 (laboratory staff) subsequently remained at EBC for the duration of the expedition (n = 10), whilst Group 2 (climbers; n = 14) subsequently ascended above 5,300 m, to a maximum of 8,848 m on Mount Everest. Measurements were repeated in both groups at EBC (5,300 m) at week 6 and week 8. Ambient temperatures were well controlled during testing [16].

### Plasma Biomarker Analyses

#### Blood sampling

All blood samples were taken prior to exercise testing and drawn from the antecubital vein. Plasma was separated from blood cells by centrifugation of whole blood at 800×g for 15 min and immediately frozen in 1 ml aliquots in liquid nitrogen. Samples were stored in liquid nitrogen for the duration of the expedition (including carriage back to Kathmandu), transported back to the UK on dry ice, then stored at −80°C in a commercial cryostorage facility until analysis. A summary of the number of samples available for each biomarker is reported in **Table S2 in File S1**.

#### Peripheral oxygen saturation

SpO_2_ was measured in the morning of the same day that blood was analyzed, after 10 minutes of rest, using a pulse oximeter (Onyx 9500, Nonin, USA). Measurements were taken by independent individuals, with the subject blind to oximeter data (thus preventing feedback to alter respiratory rate, and hence SpO_2_).

#### Oxidative Stress Markers

Isoprostanes (8-iso-prostaglandin F-2α) and 4-hydroxy-2-nonenal (4-HNE) were quantified using direct competitive enzyme immunoassay (Assay Designs, Ann Arbor, MI) and ELISA (OxiSelect HNE-His Adduct ELISA kit, Cell Biolabs, San Diego, CA), respectively. Reduced and oxidized glutathione were determined spectrofluorimetrically (DetectX, Arbor Assays, Ann Arbor, MI). For methodological reasons, this assay overestimates oxidized glutathione; thus reported values should not be misinterpreted to reflect true circulating concentrations.

#### Inflammatory Markers

Interleukin-6, TNF-α and MIF were quantified using xMAP technology (Human-Cytokines-12-plex-panel, BioRad) on a Luminex/Bio-Plex-200 System with high-throughput fluidics (BioRad).

#### Glucose, Insulin and C-Peptide

Plasma glucose levels were measured with the glucose oxidase method. Insulin and C-Peptide levels were determined using the Bio-Plex-Pro-Human-Diabetes 12-plex panel (BioRad).

#### Counter-regulatory hormones

Glucagon was measured with the Bio-Plex-Pro-Human-Diabetes 12-plex panel (BioRad). Adrenalin and Noradrenalin were quantified using a direct competitive enzyme immunoassay (Bi-CAT ELISA, Alpco Diagnostics, Salem, NH).

#### Lactate, Creatinine and Osmolality

Lactate and creatinine concentrations were determined colorimetrically by coupled enzymatic reactions using commercial assay kits (Sigma). Plasma osmolality was measured using freezing point depression (Model 3320 Micro-Osmometer, Advanced Instruments, Inc).

#### Assessment of insulin resistance

The degree of insulin resistance was estimated by the homeostasis model assessment of insulin resistance (HOMA-IR) and the fasting glucose/insulin ratio (FGIR). HOMA-IR was computed as follows: fasting insulin (µIU/ml) × fasting glucose (mmol/ml)/22.5 [29]. C-Peptide is a fragment cleaved from pro-insulin and secreted in amounts equimolar to those of insulin [31]. The longer half-life and the minimal hepatic first-pass extraction compared to insulin support the utilisation of C-Peptide as a measure of β-cell secretion [32]. The fasting C-peptide–to–insulin ratio was calculated as a measure of hepatic insulin extraction (C-peptide–to–insulin ratios decrease as hepatic insulin extraction decreases) [30].The C-Peptide/Insulin ratio can be used as a surrogate measure of hepatic insulin clearance which is correlated with hepatic insulin effects [33]. The glucagon-insulin ratio was calculated as an index of the pancreatic endocrine response [34].

### Statistical Analysis

Variables were inspected for normality of distribution using the Q-Q and the Shapiro-Wilk tests, and appropriate transformations were utilised (where necessary) to normalise the data before statistical analysis. However, we also reported untransformed values so as to provide more interpretable information on the changes of the various biomarkers; median and inter-quartile range (25th and 75th percentile) were used as descriptive statistics. A general linear model for repeated measures was used to analyse changes in biomarker concentrations during the expedition. The Bonferroni post-hoc test was used to evaluate significant changes relative to baseline (London). The method proposed by Bland and Altman was utilised to calculate within-subject coefficients of correlation to evaluate significant associations between variables [35]. Multiple-testing was taken into account and the criterion for statistical significance was therefore set at a p value <0.001. Analyses were conducted using SPSS-19 for Windows and Sigmaplot-11 for Windows.

## Results

### Baseline Characteristics and Weight Changes

Twenty-four subjects participated in the expedition; 2 subjects were excluded because biomarkers data were missing, leaving data from 22 subjects (17 males) included in the final analysis. Subjects were stratified according to their main task during the expedition: Group 1 (EBC laboratory staff; n = 10) and Group 2 (climbers; n = 12). The two groups were well matched for BMI and circulating levels of biomarkers involved in glycaemic control, indices of insulin resistance, inflammation (IL-6, CRP, TNF-α, MIF), oxidative stress and SpO_2_. While glucose levels were within the normal range, levels of fasting insulin, C-Peptide and HOMA-IR exceeded expected physiological levels (**Table 1**). Subjects lost body weight during the expedition (-7.3±5.0 kg, p<0.001), with climbers losing more weight than EBC residents (−9.0 kg vs −4.3 kg respectively, p = 0.03) (**Figure S1 in File S1**). The average rate of weight loss during the expedition was 173 g/day.

**Table 1:** Baseline characteristics of the CXE 2007 Expedition Team and comparison between team members reaching the summit and members residing at Everest Base Camp during the 8-week expedition.

	All	Climbers	Everest Base Camp	p
N	22	12	10	-
Gender (M/F)	17/5	11/1	6/4	-
Age, years	34.05 (24.02, 40.62)	36.15 (31.35, 40.67)	30.85 (25.37, 42.25)	NS
Weight, kg	76.0 (66.8, 83.8)	79.5 (70.0, 89.0)	71.0 (63.8, 80.3)	<0.05
Height, cm	178.8 (171.5, 181.0)	180.0 (176.4, 182.1)	174.0 (165.5, 180.3	<0.05
BMI, kg/m^2^	24.40 (22.82, 26.07)	24.90 (22.82, 26.37)	23.65 (22.65, 24.92)	NS
Glucose, mM/L	4.50 (3.80, 4.90)	4.34 (3.55, 4.84)	4.64 3.85 4.97	NS
Insulin, mU/mL	29.38 (26.55, 34.26)	30.52 (27.51 37.10)	27.91 24.09 32.48	NS
C-peptide, pg/mL	1590.53 (1353.74, 1819.91)	1726.26 (1483.78 1945.38)	1403.26 1302.69 1617.22	NS
HOMA-IR	5.46 (4.56, 6.38)	5.36 (4.61, 6.87)	5.53 (3.92, 6.34)	NS
FIGR	2.65 (2.03, 3.39)	2.23 (1.90, 3.10)	3.20 (2.15, 3.41)	NS
C-Peptide/Insulin Ratio	1.28 (1.20, 1.43)	1.33 (1.21, 1.58)	1.24 (1.12, 1.32)	NS
Glucagon (pg/mL)	339.06 (307.81, 392.21)	351.89 (314.00, 406.17)	334.69 (294.22, 368.83)	NS
Adrenalin (ng/mL)	0.02 (0.008, 0.05)	0.01 (0.003, 0.04)	0.02 (0.01, 0.05)	NS
Noradrenalin (ng/mL)	0.13 (0.07, 0.16)	0.14 (0.10, 0.17)	0.09 (0.06, 0.16)	NS
8-isoPGF, ng/mL	36.20 (28.64, 47.38)	33.94 (29.34, 40.79)	43.45 (27.64, 47.92)	NS
4-HNE, mg/mL	108.22 (91.54, 141.28)	105.72 (91.08, 154.09)	110.72 (97.24, 134.77)	NS
GSH, µM^¥^	0.53 (0.47, 0.57)	0.53 (0.44, 0.56)	0.53 (0.47, 0.60)	NS
GSSG, µM^¥^	0.20 (0.07, 0.29)	0.17 (0.03, 0.25)	0.27 (0.14, 0.45)	NS
GSH/GSSG^¥^	2.07 (1.15, 3.98)	2.29 (1.14, 5.84)	1.87 (1.14, 2.77)	NS
Total Glutathione, µM^¥^	0.91 (0.68, 1.13)	0.88 (0.52, 1.03)	1.07 (0.89, 1.50)	NS
IL-6, pg/mL	11.75 (10.11, 14.90)	11.31 (10.04, 15.52)	12.07 (10.13, 14.15)	NS
CRP, ng/mL	626.6 (306.2, 2085.4)	480.8 (305.0, 1614.9)	670.08 (452.0, 3043.1)	NS
TNF-α, pg/mL	31.31 (27.53, 38.92)	32.89 (27.65, 38.92)	28.85 (26.14, 36.21)	NS
MIF (pg/mL)	6714.3 (5503.8, 7047.8)	6806.9 (5816.2, 7260.7)	6316.3 (5306.1, 7004.8)	NS
Lactate (mmol/L)	1.30 (1.22, 1.64)	1.35 (1.11, 1.60)	1.27 (1.12, 1.79)	NS
SpO_2_ (%)	97.0 (98.0, 99.0)	97.0 (98.0, 99.0)	97.0 (98.0, 99.0)	NS

Data are presented as median (25th and 75th percentile). N =  number of subjects; M =  Male; F =  Female; BMI =  body mass index; HOMA-IR =  homeostasis model of assessment-insulin resistance; 8-isoPGF =  8-iso-Prostaglandin F_2_alpha; 4-HNE  =  4-Hydroxynonenal; GSH =  reduced glutathione; GSSG =  oxidized glutathione; IL-6 =  interleukin 6; CRP =  C-reactive protein; TNF-α =  tumour necrosis factor alpha; MIF =  macrophage migration inhibitory factor; SpO_2_ =  peripheral oxygen saturation. Data were transformed before analysis. T test was used to determine differences between the two groups. NS =  not statistically significant. ^¥^Data for these biomarkers were not available from the samples collected in London; therefore we have used the data derived from the samples collected in Kathmandu as baseline levels.

### Evidence of Hypoxemia, and Lactate, Osmolality and Creatinine Changes

During ascent, median peripheral oxygen saturation (SpO_2_) fell from 98.0% at sea level to 82.0% on arrival at 5,300 m (p<0.001) and improved marginally with time spent at that altitude (87.0% on EBC day 71; **Table S1 in File S1**). SpO_2_ measured at EBC was not different between climbers and EBC team during the entire expedition.

Baseline lactate levels were within the normal range (1.39±0.42 mmol/L). They progressively increased during the ascent, peaked at EBC week 1 (2.12±0.89 mmol/L; p<0.001) and remained elevated at EBC week 6 (1.78±0.53 mmol/L; p<0.05) and week 8 (1.89±0.48 mmol/L; p<0.05). Osmolality did not change during the expedition (Baseline: 292.9±5.8 mOsm/kg; EBC week 8: 291.1±12.5 mOsm/kg; p>0.05). Creatinine levels were unmodified until EBC week 6 but showed a significant increase in both groups at the end of the study (baseline: 0.82±0.11 mg/dl; EBC week 8: 1.03±0.16 mg/dl, p<0.001).

### Hypoxemia and Gluco-Insular Homeostasis

**Figure 1:**
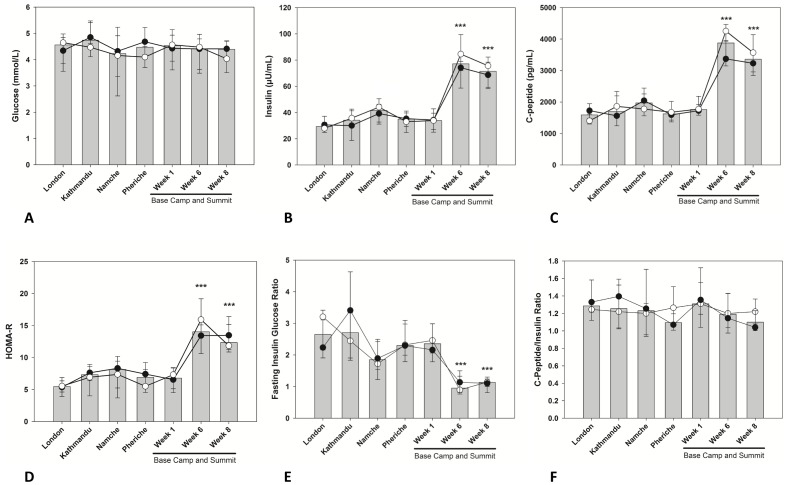
Gluco-insulin axis regulation in members of the research team reaching the summit (•) or remaining at base camp (○) during the expedition. Homeostatic model of assessment was calculated as a proxy indicator of systemic insulin resistance (HOMA-IR). C-Peptide/Insulin Ratio was calculated as a measure of hepatic insulin extraction. Median data are presented for the two groups (lines) and all subjects (bars). Error bars are 25th and 75th percentiles. Significant differences were flagged in each graph: ***p<0.001. No significant differences were observed between climbers and base camp at either time point.

Glucose levels were maintained within the normal range throughout the expedition and no differences were observed between the two groups (**Figure 1a**). Low glucose levels (<3 mmol/L) were mostly observed during the ascent phase (6 measurements) whereas only one low value was observed at high altitude (EBC week 6, climbers). A different pattern was observed for insulin axis responses. Insulin levels increased slightly during the ascent and returned to baseline values at the end of the first week at EBC. However, fasting insulin levels increased by 250% after 6 weeks at EBC (p<0.001) and this was maintained until the end of the expedition (p<0.001, EBC 8 weeks) (**Figure 1b**). Changes of comparable magnitude were observed for C-Peptide (**Figure 1c**), HOMA-IR (**Figure 1d**) and FIGR (reversed changes, **Figure 1e**). The C-Peptide/Insulin ratio appeared to be minimally affected by the prolonged exposure to hypoxia (**Figure 1f**).

### Counter-Regulatory Hormones

**Figure 2:**
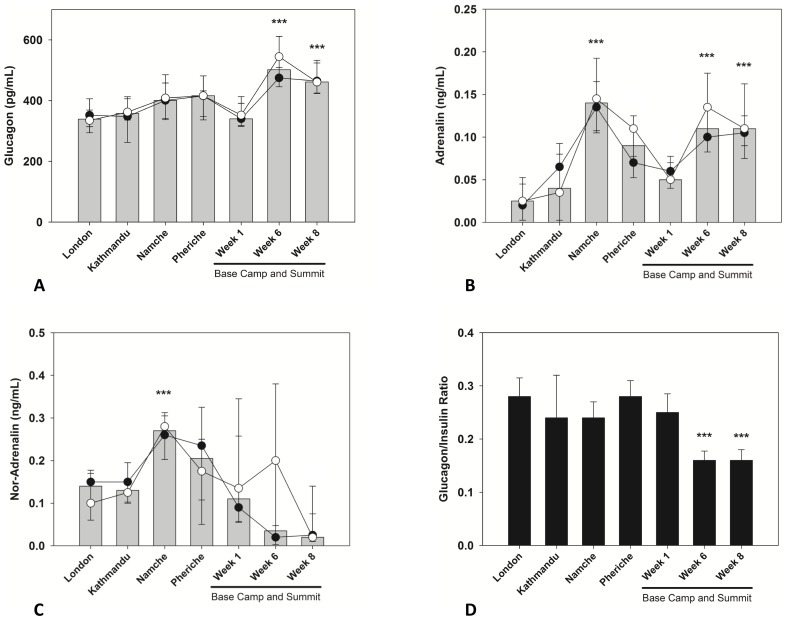
Changes in concentration of counter-regulatory hormones (glucagon, adrenalin, Noradrenalin) and glucagon-insulin ratio in members of the research team reaching the summit (•) or remaining at base camp (○) during the expedition. Median data are presented for the two groups (lines) and all subjects (bars). Error bars are 25th and 75th percentiles. Significant differences were flagged in each graph: ***p<0.001 No significant differences were observed between climbers and EBC lab team members at either time point.

Glucagon levels had risen significantly by week 6 (p<0.001) and week 8 (p<0.001), albeit to a lesser proportional extent than those of insulin and C-peptide. No differences in the responses were found between climbers and EBC residents (**Figure 2a**). One week after arrival at EBC, Adrenalin and Noradrenalin concentrations changed in opposite directions, and the response to sustained hypoxia was greater for Adrenalin (**Figure 2b**) compared to Noradrenalin (**Figure 2c**). The glucagon-insulin ratio decreased significantly during the last two weeks of the expedition (p<0.001) (**Figure 2d**).

### Biomarkers of Oxidative Stress

**Figure 3:**
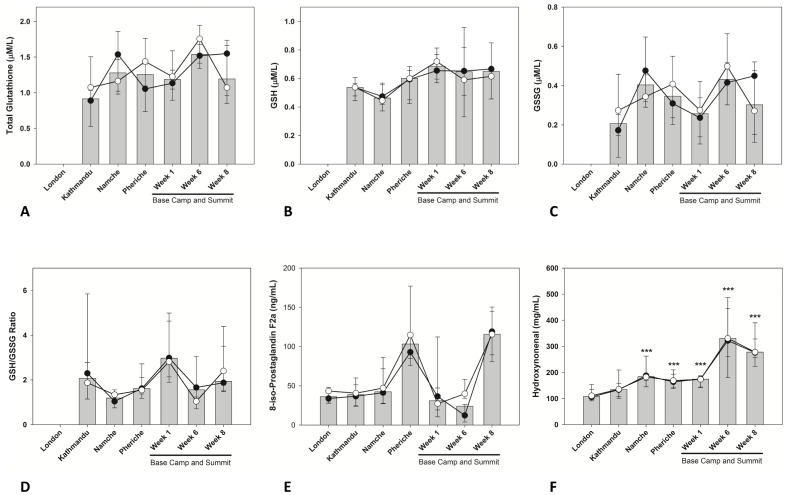
Changes in biomarkers of oxidative stress in members of the research team reaching the summit (•) or remaining at base camp (○) during the expedition. Median data are presented for the two groups (lines) and all subjects (bars). Error bars are 25th and 75th percentiles. Data for Total Glutathione, GSH, GSSG and GSH/GSSG were not available from the samples collected in London. Therefore we have used the data derived from the samples collected in Kathmandu as baseline levels. Significant differences were flagged in each graph: ***p<0.001 No significant differences were observed between climbers and EBC lab team members at either time point.

We have previously reported some of these data [36]. Levels of total glutathione and related biomarkers (GSH, GSSG, GSH/GSSG) were compared to the values taken at the start of the ascent (Kathmandu, 1,300 m), due to unavailability of baseline measures for London. responses varied substantially between individuals, with a non-significant trend for total glutathione and GSH to increase at high altitude (**Figure 3a**–**d**). Marked inter-individual variation in Isoprostane levels (8-isoPGF) was also identified, with a complex pattern of response to ascent (**Figure 3e**). By contrast, 4-HNE showed much smaller inter-individual variability and a direct association with the duration of hypoxia. Changes in circulating levels were significantly elevated during the ascent and had increased almost 3-fold by EBC week 6 and week 8 (p<0.001) (**Figure 3f**).

### Biomarkers of Inflammation

**Figure 4:**
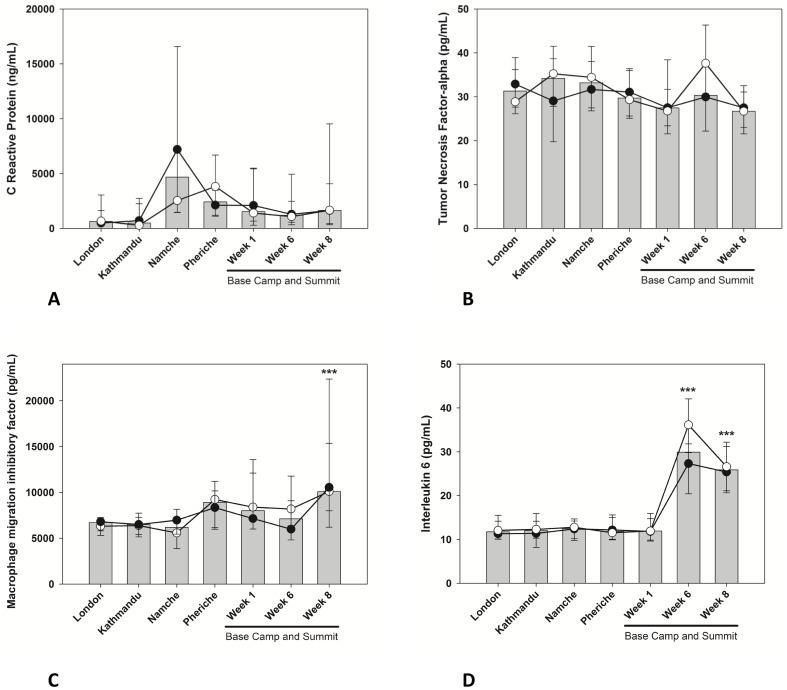
Changes in biomarkers of inflammation in members of the research team reaching the summit (•) or remaining at base camp (○) during the expedition. Median data are presented for the two groups (lines) and all subjects (bars). Error bars are 25th and 75th percentiles. Significant differences were flagged in each graph: ***p<0.001. No significant differences were observed between climbers and EBC lab team members at either time point.

CRP levels did not change significantly (**Figure 4a**) whilst TNF-α levels showed a tendency to decline (**Figure 4b**). MIF increased only at EBC week 8 (p<0.001) (**Figure 4c**) whereas the profile of IL-6 mirrored changes in insulin levels. Specifically, IL-6 levels showed no changes until after EBC week 1 but had increased by ∼250% by EBC week 6 and week 8 (p<0.001) (**Figure 4d**).

### Correlation Analysis

**Figure 5:**
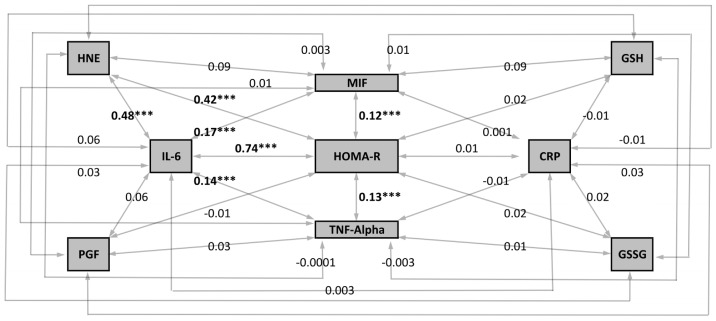
Correlation between Homeostatic Model of Assessment of insulin resistance (HOMA-IR) with biomarkers of inflammation and oxidative stress during the 2007 Caudwell Xtreme Everest Expedition. The pathogenic model is centripetal (direction of causality is towards the centre of the diagram) implying that the position of the biomarkers reflects the hypothesised order of activation of the responses to hypoxia (Oxidative Stress → Inflammation → Insulin Resistance (HOMA-IR)). Within-subject correlation analysis was performed and correlation coefficients are reported. Significant results have been highlighted: ***p<0.001. Acronyms: 4-HNE  =  4-Hydroxynonenal; PGF =  8-iso-Prostaglandin F_2_alpha; IL-6 =  interleukin 6; MIF =  macrophage migration inhibitory factor; GSH =  reduced glutathione; TNF-α =  tumour necrosis factor alpha; CRP =  C-reactive protein; GSSG =  oxidized glutathione.

The main aim of this analysis was to evaluate the associations between hypoxemia, oxidative stress and inflammatory biomarkers with glucose, insulin and indices of insulin resistance. A correlation matrix has been provided in **Table S3 in File S1**. In addition, a mechanistic-model of insulin resistance based on the correlation of HOMA-IR with biomarkers of inflammation and oxidative stress has been proposed (**Figure 5**). The model suggests a non-significant role of CRP whereas a strong and significant association with HOMA-IR was observed for IL-6 (r = 0.74, p<0.001). The correlation of oxidative stress with inflammation revealed an important relationship between IL-6 with 4-HNE (r = 0.48, p<0.001). 4-HNE was the only oxidative stress biomarkers strongly correlated with HOMA-IR (r = 0.42, p<0.001) (**Figure 5**). The strongest association with insulin and indices of gluco-insular regulation were observed for glucagon, IL-6 and 4-HNE. SpO_2_ was weakly associated with biomarkers of insulin resistance (**Table S3 in File S1**).

## Discussion

The key finding of the present study is that sustained hypoxia induced a significant increase in IL-6 and 4-HNE concentrations which were correlated with insulin concentrations and HOMA-IR levels.

### Gluco-Insular Response

Our results are in accord with those from the (hypobaric chamber-based) simulated ascent of Everest in Operation Everest II, whose study duration (40 days) and intensity of exposure to hypoxia was similar for the 6 healthy male participants to that which our subjects faced [12]: in both studies, glucose- insulin response was dependent on the duration and intensity of exposure to hypoxia, with a paradoxical increase in insulin levels during weight loss [14]. These data are consistent with an increase in insulin resistance at altitude, resulting in a compensatory increase in insulin secretion. Such an increase in insulin levels may also help compensate for the increase in hepatic glucose output induced by counter-regulatory hormones (glucagon, adrenalin), whose levels rose by ∼70% and ∼300%, respectively during the last two weeks at altitude.

Results from other studies testing the association between hypoxia and insulin function are conflicting. Short-term exposure to hypoxia for two days at high altitude was characterised by increased glucose and insulin levels which progressively returned to baseline levels within one week [3]. Simulated short-term (16 hours) exposure to hypoxia (4,300 m) induced a decline in insulin sensitivity in both women and men [37]. Endocrine responses to hypoxia have also been investigated in longer studies. Plasma glucose and insulin levels increased in fifteen healthy subjects after seven days at 5,080 m but returned to baseline values at the end of the 41-day expedition. Decreased glycaemia and stable insulin levels were observed in nine male elite climbers during their ascent of Mt. Everest [1].

The Operation Everest II study resembles the Caudwell Xtreme Everest Expedition for study duration and intensity of exposure to hypoxia. Weight loss in the 6 healthy men making a simulated (hypobaric chamber) ascent of Mt. Everest over 40days [12] was similar to that seen in our study (-7.4 kg). This study reported a two-fold rise of insulin levels with ascent to 335Torr (25days; altitude equivalent = ∼5700 m) and 282Torr (32days, altitude equivalent = ∼7000 m) that remained elevated until the end of the study (40days, 282Torr). Plasma glucose concentrations were unchanged throughout the study [12]. Operation Everest II did not, however, report significant changes in either glucagon or adrenalin concentrations [12]. Larsen et al measured glucagon and adrenalin levels in healthy subjects after seven days at high altitude and found no change in the blood concentrations of either hormone [3]. In our study, Noradrenalin concentrations peaked as soon as subjects had to face the first substantial environmental stressor represented by the ascent to Namche Bazaar at the start of the trekking expedition [16]. Subsequently, Noradrenalin levels progressively declined, returning to baseline levels during the last two weeks of the expedition. A similar pattern for Noradrenalin concentrations was observed in the Operation Everest II study [14]. The physiological meaning of the reciprocal changes in adrenalin and Noradrenalin concentrations at the end of the study is not clear. Perhaps the elevated metabolic stress occurring during the last part of the expedition may have posed a greater demand on systemic functions (metabolic, respiratory) regulated by adrenalin and, therefore, a reduction of the adrenalin/Noradrenalin ratio may have enhanced these responses.

The role of hepatic insulin resistance and neo-glucogenesis had been previously investigated in healthy men exposed to 7-day high altitude hypoxia (4,559 m) utilising hyperglycaemic euglycaemic clamps to determine peripheral and hepatic insulin resistance [3]. Insulin action decreased after two days of hypobaric hypoxia but improved with more prolonged exposure. No effects of hypoxia were observed on hepatic gluconeogenesis [3]. Glucose and insulin levels were unchanged during the ascent and the first week at EBC, suggesting that a longer exposure to hypoxia is needed to induce changes in insulin signalling.

The effects of prolonged hypoxia exposure on gluco-insular regulation may be contextualised within a pathogenetic allostatic metabolic model [38]. Chronic hypoxia represents the allostatic metabolic load, which initiates a series of adaptive responses leading to maintenance of physiological function (allostasis). Over time, the allostatic hypoxic load can accumulate and activate neural, endocrine, and immune stress responses which in turn modify glucose and insulin regulation. This concept has been previously proposed to explain the pathogenesis of hyperglycemia and insulin resistance [39].

### Changes in 4-HNE and IL-6

The uniqueness of our results is in the identification of a consistent association between 4-HNE, IL-6 and insulin resistance (i.e., HOMA-IR), which suggests the existence of a mechanistic link between these molecules and the development of insulin resistance during exposure to hypoxia. The two oxi-inflammatory biomarkers (4-HNE and IL-6) may represent the stress mediators of chronic exposure to hypoxia. In addition, the associations appeared to be minimally affected by changes in energy balance as the oxidative-inflammatory and glucose-insular responses to hypoxia were similar in both groups, despite the significant differences in weight changes and physical activity between groups during the last two weeks of the expedition. These changes coincided temporally with the increases in insulin concentrations and HOMA-IR. IL-6 also showed a strong correlation with glucagon, which suggests that the mechanistic link between the prolonged exposure to hypoxia and pancreatic endocrine function may be modified by IL-6.

This study is the first to report the association between sustained hypoxia, IL-6 and gluco-insular hormones. Hartmann et al reported increased IL-6 levels in healthy volunteers who spent 3 nights at an elevation above 3,400 m [40]. However, this acute response was not observed in our study. IL-6 is secreted by enlarged, activated adipocytes which may result in a systemic elevation of IL-6 plasma levels leading to insulin resistance [41]. In muscular tissue, IL-6 may be secreted during physical activity to act as an energy sensor by activating AMP-kinase and enhancing glucose disposal. The evidence suggests an association between chronic elevations of IL-6 and hepatic insulin resistance. IL-6 also appears to impede maturation of pre-adipocytes and deregulate the insulin pathway [42]. Therefore, the association of IL-6 with insulin function is still controversial; our results suggest that, under chronic hypoxic conditions, IL-6 may be influencing the physiology of pancreatic endocrine hormones. However, the causality of the associations and the physiological mechanisms underpinning these associations remain to be established. Pedersen et al have summarised the role of IL-6 as a metabolic modulator of muscular function during exercise [43]. IL-6 concentrations scaled with exercise duration and intensity but concentrations returned to basal levels shortly after cessation of exercise [43]. The investigators concluded that IL-6 plays a physiological role during muscle contraction as it seems to have anti-inflammatory effects and a role in fat oxidation, and improved insulin-stimulated glucose uptake [43]. The metabolic role of IL-6 cannot be ignored in the interpretation of our results. However, the effects of muscle contraction on circulating IL-6 concentrations are likely to have been limited because 1) samples were collected in the morning after subjects had rested overnight, and 2) plasma IL-6 levels of base camp residents and climbers were overlapping in spite of very different physical activity levels during the last part of the expedition.

Hypoxia is associated with mitochondrial dysfunction and production of ROS [44]. This is also caused by a reduced capacity of antioxidant systems leading to increased oxidized glutathione concentrations and a decline in mitochondrial SOD activity/content [45]. Increased ROS are associated with oxidative damage to lipids, proteins, and DNA [46]. Oxidative stress levels seem to be linearly related to altitude, degree of hypoxia and duration of exposure. The Operation Everest III study suggested a direct association as lipid peroxidation increased by 23% at 6,000 m and 79% at 8,848 m [47]. Vij *et al* studied acclimatization to high-altitude hypoxia over a period of 13 months, and it appeared that a period of 3 months was sufficient to increase lipid peroxidation and decrease enzymatic and non-enzymatic antioxidant defences [48]. In our study, hypoxia induced progressive changes in biomarkers of oxidative stress. In particular, 4-HNE levels increased significantly during the last phase of the expedition and, interestingly, the inter-subject variability of these responses was relatively low (CV%: ∼45%) compared to isoprostanes (CV%: ∼110%) and reduced glutathione (CV%: ∼300%). ROS production could have a signalling role by communicating the declined oxidative efficiency of the cells and thereby facilitate glucose uptake through insulin-independent GLUT-1 [49], [50]. The reduction of insulin sensitivity and activation of anaerobic oxidative pathways could therefore represent an energy-buffer mechanism to reroute glucose and oxygen towards vital organs such as brain and heart. Increased insulin secretion during hypoxia may therefore be a reflection of the increased reliance of cellular metabolism on glycolysis [26].

### Limitations

Subjects were exposed to other extreme environmental conditions (low temperature, physical exertion) in addition to hypoxia which may confound the associations with metabolic outcomes. However, although climbers experienced much harsher conditions than EBC residents, they did not show higher levels of oxidative stress, inflammation or insulin levels. This suggests that metabolic derangements were essentially driven by the prolonged exposure to hypoxia. We were unable to determine the exact time of onset of the metabolic derangements as no measurements were taken between week 1 and week 6 at EBC. Cortisol levels were not measured during the expedition and any possible association with changes in insulin and glucagon concentrations cannot therefore be assessed. Finally, subjects were initially of normal weight and thence in negative energy balance, which may limit the transferability of the results to obese subjects in positive energy balance as a mechanistic model of insulin resistance. However, in consideration of the established insulin sensitising effects of weight loss we argue that this study supports the role of systemic hypoxia in the impairment of gluco-insular action as well as defines a potential pathogenetic trajectory that may link tissue hypoxia to insulin resistance in obese subjects.

## Conclusions

Healthy humans exposed to sustained hypobaric hypoxia at high-altitude appear to become insulin-resistant while they are in negative energy balance. Normo-glycemia was maintained by a compensatory increase in β-cell secretion and reduced hepatic extraction leading to sustained hyperinsulinemia during the last two weeks of the expedition. Previous cellular and animal models of hypoxia-induced insulin resistance have identified two core causal mechanisms: over-production of ROS and inflammation [22]. Our results are aligned with this mechanistic paradigm and provide evidence to support a causal relationship between hypoxia and insulin resistance in humans. The role of 4-HNE and IL-6 as mediators of these effects should be corroborated in future studies. Ideally, these studies should be conducted in simulated-hypoxic environments where other factors can be constrained to control for the confounding effects of weight loss while utilising dedicated experimental protocols for the assessment of peripheral and hepatic insulin resistance.

## Supporting Information

File S1 **Supporting information figure and tables. Figure S1,** **Cumulative mean changes in body weight occurred at the end of the Caudwell Everest Expedition in climbers and in members residing at base camp (EBC).** Mann-Whitney test was used to determine between-group differences in weight loss after EBC 8 weeks. **Table S1,** Ascent profile and residence at high altitude of the team remaining at Everest Base Camp (EBC, N = 10) and those climbing higher (Climbers, N = 14)*. Information on altitude (m), barometric pressure (mmHg) and inspired partial pressure of oxygen (PiO_2_, mmHg) is reported for each phase. Hemoglobin concentrations (g/dl) and arterial oxygen saturation (SaO_2_, %) are reported in each group during the different phases of the expedition. **Table S2, Summary of the number of samples included in the analyses for each biomarker. Table S3, Correlation matrix to look at association between biomarkers of inflammation, oxidative stress and counter-regulatory hormones with glucose, insulin, C-peptide and HOMA-R.**
(DOCX)
