# Supplementary material for: Effects of Prolonged Exposure to Hypobaric Hypoxia on Oxidative Stress, Inflammation and Gluco-Insular Regulation: The Not-So-Sweet Price for Good Regulation
Source: PLoS One. 2014 Apr 14;9(4):e94915. doi: 10.1371/journal.pone.0094915 (PMC3986261; doi:10.1371/journal.pone.0094915)
Supplement: File S1 — Supporting information figure and tables. Figure S1, Cumulative mean changes in body weight occurred at the end of the Caudwell Everest Expedition in climbers and in members residing at base camp (EBC). Mann-Whitney test was used to determine between-group differences in weight loss after EBC 8 weeks. Table S1, Ascent profile and residence at high altitude of the team remaining at Everest Base Camp (EBC, N = 10) and those climbing higher (Climbers, N = 14)*. Information on altitude (m), barometric pressure (mmHg) and inspired partial pressure of oxygen (PiO2, mmHg) is reported for each phase. Hemoglobin concentrations (g/dl) and arterial oxygen saturation (SaO2, %) are reported in each group during the different phases of the expedition. Table S2, Summary of the number of samples included in the analyses for each biomarker. Table S3, Correlation matrix to look at association between biomarkers of inflammation, oxidative stress and counter-regulatory hormones with glucose, insulin, C-peptide and HOMA-R. (DOCX) [file pone.0094915.s001.docx]

**Combined Supporting Information - FILE S1**

| **Table S1: Ascent profile and residence at high altitude of the team remaining at Everest Base Camp (EBC, N=10) and those climbing higher (Climbers, N=14)*. Information on altitude (m), barometric pressure (mmHg) and inspired partial pressure of oxygen (PiO_2_, mmHg) is reported for each phase. Hemoglobin concentrations (g/dl) and arterial oxygen saturation (SaO_2_, %) are reported in each group during the different phases of the expedition.** | | | | | | | | |
| --- | --- | --- | --- | --- | --- | --- | --- | --- |
|  |  | **Phase/Location** | **Altitude**  **(m)** | **Barometric Pressure**  **(mmHg)** | **PiO_2_**  **(mmHg)** | **Hb**  **(g/dl)** | **SaO_2_**  **(%)** | **Plasma Samples for**  **Biomarker Assessment** |
| **Ascent to EBC** | **Day 0**  EBC lab team  Climbers | Baseline (London) | 75 | 754 | 148.0 | 13.3 (0.8)  14.2 (0.7) | 98.0 (0.8)  97.9 (1.1) | √ |
|  | **Day 1**  EBC lab team  Climbers | Kathmandu | 1300 | 650 | 126.2 | 14.2 (0.8)  14.8 (0.8) | 96.6 (1.7)  95.9 (1.3) | √ |
|  | **Day 3**  EBC lab team  Climbers | Namche | 3500 | 505 | 95.4 | 14.7 (0.7)  16.0 (1.1) | 91.0 (5.7)  90.6 (2.1) | √ |
|  | **Day 7**  EBC lab team  Climbers | Pheriche | 4250 | 461 | 86.7 | 15.0 (1.1)  15.8 (0.8) | 87.0 (4.4)  87.4 (2.3) | √ |
|  | **Day 12**  EBC lab team  Climbers | Arrival at EBC | 5300 | 404 | 74.7 | - | - | - |
| **High Altitude** | **Day 19 (Week 1)**  EBC lab team  Climbers | EBC | 5300 | 404 | 74.7 | 16.9 (1.7)  17.9 (1.5) | 77.1 (6.3)  82.6 (4.1) | √ |
|  | **Day 38 (Week 3)**  EBC lab team  Climbers | EBC  Camp 2 | 5300  6400 | 404  350 | 74.7  63.4 | 17.7 (1.1)  19.2 (2.0) | 84.1 (5.0)  75.9 (6.4) | - |
|  | **Day 56 (Week 6)**  EBC lab team  Climbers | EBC | 5300 | 404 | 74.7 | 17.3 (1.7)  18.5 (1.1) | 82.8 (5.4)  85.6 (3.9) | √ |
|  | **Day 62 (Week 7)**  EBC lab team  Climbers | EBC  Summit | 5300  8848 | 404  253 | 74.7  42.8 | - | - | - |
|  | **Day 70 (Week 8)**  EBC lab team  Climbers | EBC | 5300 | 404 | 74.7 | 17.7 (1.4)  19.4 (1.2) | 84.3 (8.1)  87.2 (3.8) | √ |

*Fourteen subjects participated in this part of the expedition of whom 8 reached the summit of Everest; 2 subjects were excluded because biomarkers data were missing and therefore 12 subjects from this group were included in the final analysis. All subjects followed an identical ascent profile during the ascent to EBC and, to standardize hypoxic exposure, were prevented from excursions of more than 300 m from the group altitude at any time. For Group 1 (BC laboratory staff), excursions were limited to within 500 vertical metres of the BC altitude for the duration of the expedition. The climbing team (n = 14) followed an identical ascent profile until the completion of all testing at Camp 2 (6,400 m) including identical acclimatisation outings. Group 1 was not exposed to supplemental oxygen for the duration of the expedition. Group 2 was not exposed to any supplemental oxygen until the completion of testing at Camp 2. All climbers used supplemental oxygen at flow rates of 2-4 l/min for the summit climb above Camp 3 (7,100 m) and at 0.5 l/min whilst sleeping at and above Camp 3. Testing was repeated at the end of the expedition (immediately prior to departure) for all climbers at EBC (days 66 to 71).


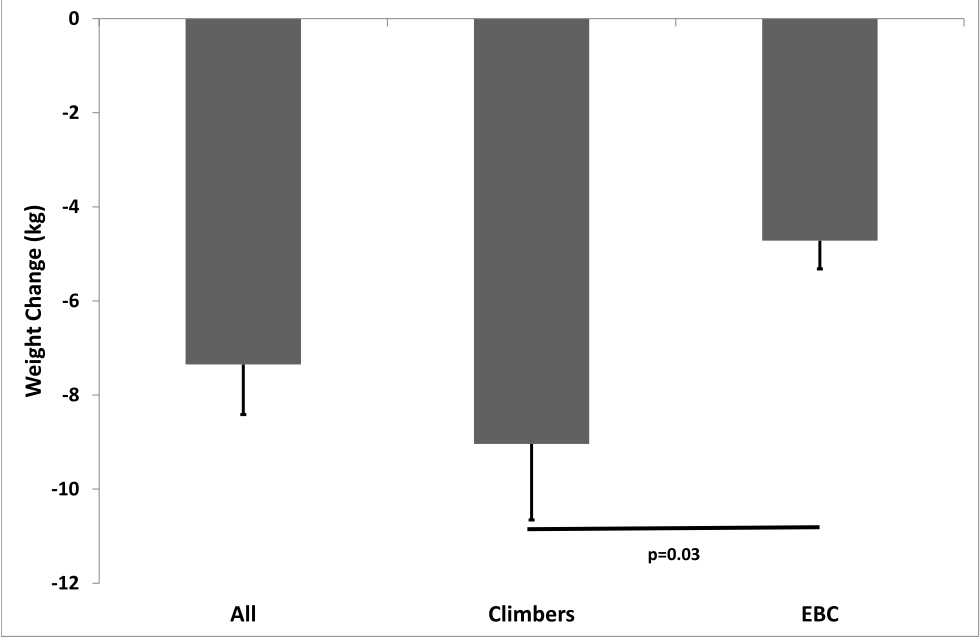


**Figure S1**: Cumulative mean changes in body weight occurred at the end of the Caudwell Everest Expedition in climbers and in members residing at base camp (EBC). Mann-Whitney test was used to determine between-group differences in weight loss after EBC 8 weeks.

| **Table S2:** Summary of the number of samples included in the analyses for each biomarker | | | | | | | |
| --- | --- | --- | --- | --- | --- | --- | --- |
|  | **London** | **Kathmandu** | **Namche** | **Pheriche** | **Week1** | **Week6** | **Week8** |
| C-peptide | 22 | 22 | 22 | 22 | 22 | 20 | 21 |
| Insulin | 22 | 22 | 22 | 22 | 22 | 20 | 21 |
| Glucose | 22 | 22 | 22 | 22 | 22 | 20 | 21 |
| 8-isoPGF | 22 | 22 | 22 | 22 | 22 | 20 | 21 |
| HNE | 20 | 20 | 20 | 21 | 20 | 19 | 20 |
| GSH | 0 | 22 | 22 | 22 | 22 | 19 | 19 |
| IL-6 | 22 | 22 | 22 | 22 | 22 | 20 | 21 |
| CRP | 22 | 22 | 22 | 22 | 22 | 20 | 19 |
| TNF-alpha | 22 | 22 | 22 | 22 | 22 | 20 | 21 |
| HOMA-IR | 22 | 22 | 22 | 22 | 22 | 20 | 21 |
| GSSG | 0 | 22 | 22 | 22 | 22 | 19 | 19 |
| Total Gluthatione | 0 | 22 | 22 | 22 | 22 | 19 | 19 |
| GSH/GSSG | 0 | 22 | 22 | 22 | 22 | 19 | 19 |
| MIF | 22 | 22 | 22 | 22 | 22 | 20 | 21 |
| Lactate | 21 | 22 | 21 | 22 | 22 | 20 | 20 |
| SPO2 | 22 | 22 | 22 | 22 | 22 | 20 | 21 |
| Glucagon | 22 | 22 | 22 | 22 | 22 | 20 | 21 |
| Adrenalin | 22 | 22 | 22 | 22 | 22 | 20 | 21 |
| Noradrenalin | 22 | 22 | 22 | 22 | 22 | 20 | 21 |

| **Table S3:** Correlation matrix to look at association between biomarkers of inflammation, oxidative stress and counter-regulatory hormones with glucose, insulin, C-peptide and HOMA-R | | | | | | |
| --- | --- | --- | --- | --- | --- | --- |
|  | **Glucose** | **Insulin** | **C-peptide** | **HOMA-R** | **FIGR** | **C-Peptide/Insulin** |
| **SpO_2_** | 0.02 | **-0.13***** | **-0.15***** | -0.08 | **0.13***** | -0.002 |
| **Glucagon**  pg/mL | -0.01 | **0.80***** | **0.62***** | **0.62***** | **-0.68***** | **-0.27***** |
| **Adrenalin**  ng/mL | -0.02 | **0.16***** | 0.06 | 0.08 | **-0.18***** | -0.02 |
| **Nor-Adrenalin**  ng/mL | -0.001 | **-0.11***** | -0.10 | -0.10 | 0.07 | -0.003 |
| **8-isoPGF**  ng/mL | 0.002 | -0.003 | -0.004 | -0.001 | 0.005 | -0.001 |
| **4-HNE**  mg/mL | 0.006 | **0.52***** | **0.48***** | **0.42***** | -**0.41***** | -0.05 |
| **GSH^¥^**  μM | -0.01 | 0.04 | 0.03 | 0.02 | -0.04 | -0.02 |
| **GSSG^¥^**  μM | -0.005 | 0.04 | 0.02 | 0.02 | -0.04 | -0.03 |
| **GSH/GSSG^¥^**  μM | 0.001 | -0.02 | -0.01 | -0.01 | 0.02 | 0.01 |
| **Glutathione**  μM | -0.009 | 0.05 | 0.03 | 0.03 | -0.05 | -0.03 |
| **IL-6**  pg/mL | -0.006 | **0.90***** | **0.75***** | **0.74***** | -**0.72***** | **-0.19***** |
| **CRP**  ng/mL | -0.01 | -0.002 | -0.001 | 0.01 | 0.001 | 0.007 |
| **TNF-α**  pg/mL | -0.001 | **0.16***** | 0.04 | **0.13***** | **-0.13***** | **-0.31***** |
| **MIF**  pg/mL | -0.001 | **0.13***** | **0.12***** | **0.12***** | 0.10 | -0.01 |
| **Lactate**  (mmol/L) | 0.001 | 0.02 | 0.05 | 0.03 | -0.01 | 0.01 |

The method proposed by Bland and Altman was utilised to calculate within-subject coefficients of correlation (see methods section ). Data were transformed before analysis. Acronyms: SpO_2_= peripheral oxygen saturation (%); 4-HNE = 4-Hydroxynonenal; PGF= 8-iso-Prostaglandin F2alpha; IL-6= interleukin 6; MIF= macrophage migration inhibitory factor; GSH= reduced glutathione; TNF-Alpha= tumour necrosis factor alpha; CRP= C reactive protein; GSSG= oxidized glutathione. Significant results are highlighted in bold. Significance level is p<0.001
